# Transcripts with systematic nucleotide deletion of 1-12 nucleotide in human mitochondrion suggest potential non-canonical transcription

**DOI:** 10.1371/journal.pone.0217356

**Published:** 2019-05-23

**Authors:** Ganesh Warthi, Hervé Seligmann

**Affiliations:** 1 Aix-Marseille Université, IRD, VITROME, Institut Hospitalo-Universitaire Méditerranée-Infection, Marseille, France; 2 Aix-Marseille Université, IRD, MEPHI, Institut Hospitalo-Universitaire (IHU) Méditerranée Infection, Marseille, France; 3 The National Natural History Collections, The Hebrew University of Jerusalem, Jerusalem, Israel; John Curtin School of Medical Research, AUSTRALIA

## Abstract

Raw transcriptomic data contain numerous RNA reads whose homology with template DNA doesn’t match canonical transcription. Transcriptome analyses usually ignore such noncanonical RNA reads. Here, analyses search for noncanonical mitochondrial RNAs systematically deleting 1 to 12 nucleotides after each transcribed nucleotide triplet, producing deletion-RNAs (delRNAs). We detected delRNAs in the human whole cell and purified mitochondrial transcriptomes, and in Genbank's human EST database corresponding to systematic deletions of 1 to 12 nucleotides after each transcribed trinucleotide. DelRNAs detected in both transcriptomes mapped along with 55.63% of the EST delRNAs. A bias exists for delRNAs covering identical mitogenomic regions in both transcriptomic and EST datasets. Among 227 delRNAs detected in these 3 datasets, 81.1% and 8.4% of delRNAs were mapped on mitochondrial coding and hypervariable region 2 of dloop. Del-transcription analyses of GenBank's EST database confirm observations from whole cell and purified mitochondrial transcriptomes, eliminating the possibility that detected delRNAs are false positives matches, cytosolic DNA/RNA nuclear contamination or sequencing artefacts. These detected delRNAs are enriched in frameshift-inducing homopolymers and are poor in frameshift-preventing circular code codons (a set of 20 codons which regulate reading frame detection, over- and underrepresented in coding and other frames of genes, respectively) suggesting a motif-based regulation of non-canonical transcription. These findings show that rare non-canonical transcripts exist. Such non canonical del-transcription does increases mitochondrial coding potential and non-coding regulation of intracellular mechanisms, and could explain the dark DNA conundrum.

## Introduction

Raw transcriptomic data include RNA reads that do not correspond to canonical transcription of the genome [1]. The DNA template of most known noncanonical RNAs is easily recovered because their sequence usually differs from the template DNA by few single nucleotide edits. Around 80–90% of the human genome is transcribed at some point during development [2,3] and has biochemical functions [4]. Most noncanonical transcripts are noncoding RNAs (ncRNAs). Small ncRNAs are <200bp long and contribute to transcription activation [5], transcription maintenance [6], translation inhibition [7], mRNA degradation [8], gene regulation [9], epigenetic modification [10,11], and RNA polymerase II backtracking [9]. Other unknown roles might exist. These coding and non-coding RNAs in the transcriptome were detected assuming canonical transcription, and transcripts not matching template DNA were ignored in further analyses. Hence this genetic information is lost because it is not considered in the analyses of non-canonical transcription. This lost genetic information could possess answers to many questions related to evolution, genetic diseases and could explain dark DNA conundrum.

Dark DNA are hidden genes in the genome of an organism that are essential for its survival whose translational product has been detected in that organism. The Dark DNA term was first coined in the genome study of sand rat that had 87 missing genes essential for its survival. However, the functional translational product of missing genes was detected in the tissue samples, inferring possible hidden genes in sand rat’s genome [12]. Similarly, 274 genes were also reported missing in a bird’s genomes essential for its survival [13]. It is considered that the high GC content of these hidden genes affects detection by current DNA sequencing technology.

The detection of hidden genes is mainly based on the sequence similarity of RNA sequencing reads by assuming canonical transcription. Similarly, with the recent advancement in sequencing and bioinformatic technologies numerous proteins are assigned as hypothetical genes/protein whose existence is predicted but lack experimental evidences. These hypothetical proteins are predicted assuming canonical transcription followed by translation based on the identification of open reading frames in sequenced genome. However, it is very possible that many of these gene products are not canonical RNA or peptide.

RNA-DNA differences (RDDs) in the human transcriptome [14–22] due to post-transcriptional editing [23–28] and post-transcriptional hyper edited RNAs [29] explain some noncanonical transcripts. Notably, RDDs deleting some nucleotides in mitochondrial rRNAs recover more stable ancestral rRNA structures [19]. Some noncanonical transcripts result from RNA fusion [30]. In this study we are reporting short non canonical mitochondrial RNAs (called delRNAs) detected in more than one independent sequencing datasets assuming non canonical phenomenon of systematic nucleotide deletion.

Two additional types of non-canonical mitochondrial RNAs have been detected in human whole cell transcriptomes. These RNAs differ from template DNA along systematic rules. Some noncanonical RNAs result from transcription systematically exchanging nucleotides along one among 23 bijective transformations. These produce 'swinger RNA'. Nine symmetric exchanges are of type X↔Y (for example A↔C) [31–34] and fourteen asymmetric exchanges of type X→Y→Z→X (for example A→C→G→A) [34–37]. Swinger DNA has been reported until now only for one symmetric exchange, A↔T+C↔G, in organelles [38,39] and for eukaryote nucleus-encoded rRNAs [40]. Swinger RNAs were confirmed by two NGS methods (454 and SOLID) in the amoeban giant virus Mimivirus [1]. Some RNAs are chimeric: they partly correspond to regular transcription, and an adjacent stretch is transformed along a non-identical swinger transformation [41]. Peptides matching translation of such chimeric swinger RNAs (chimeric peptides) also exist [42].

**Fig 1:**
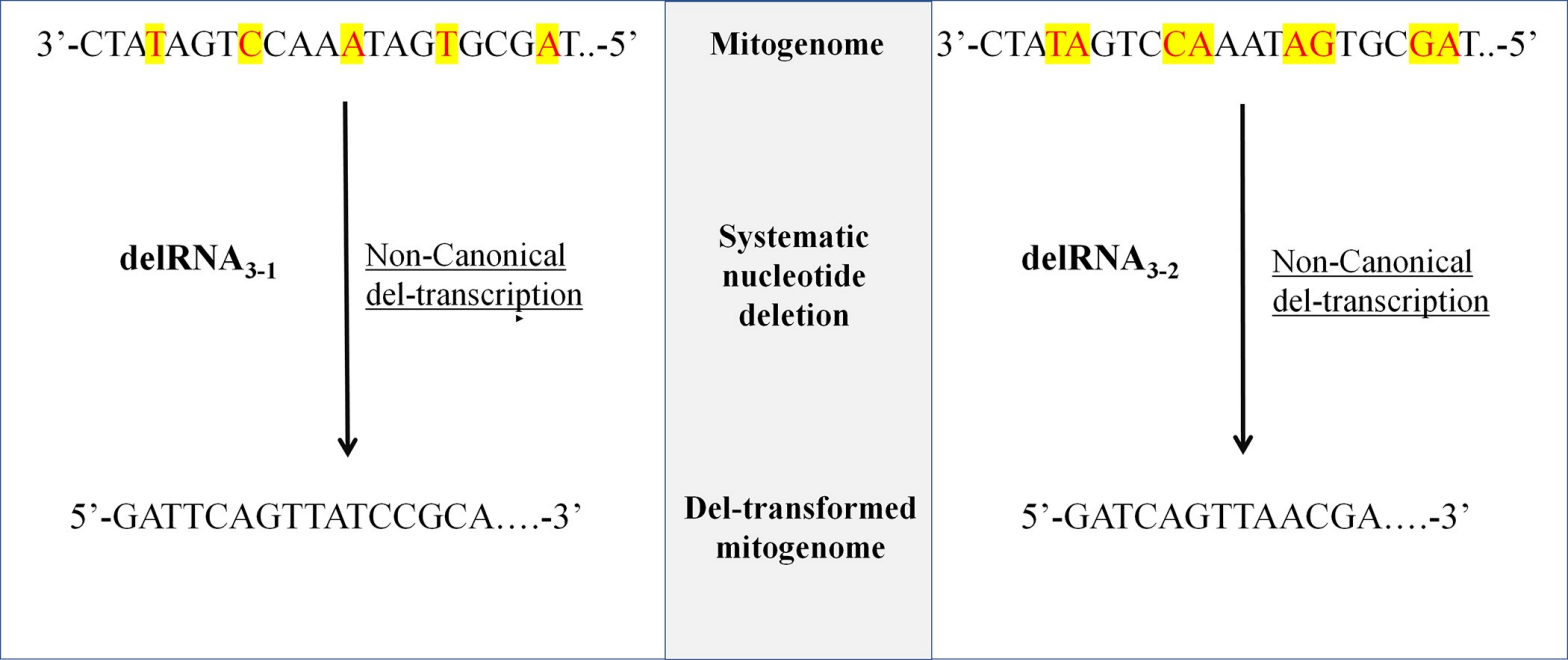
Principle of systematic deletion transcription. This principle is used for the construction of different deletion transformed versions of the mitogenome. To construct the delRNA_3-1_ transformation of the mitogenome, the 4^th^ nucleotide following each transcribed trinucleotide is deleted. Similarly, every 4^th^ and 5^th^ nucleotides are deleted in delRNA_3-2_ transformations of the mitogenome. The nucleotide(s) highlighted are the nucleotide(s) missing in del-transcribed delRNAs. These principles work for any deletion size window of k (1 to 12) nucleotides. Where k is the number of nucleotides deleted after each transcribed nucleotide triplet.

Other noncanonical RNAs result from systematic deletions of nucleotide(s) after each transcribed nucleotide triplet (NNNx), where N corresponds to transcribed nucleotides, and ‘x’ is a nucleotide missing in the RNA. Human mitochondrial RNAs with mono- (NNNxNNNxN …) and dinucleotide (NNNxxNNNxxN …) (Fig 1) deletions have been detected, and corresponding peptides were detected in mitochondrial mass spectrometry datasets by different search strategies [43,44]. These RNAs are termed delRNAs. RNAs following the pattern NNNx are indicated delRNA_3-1_, those along pattern NNNxx are delRNA_3-2_. The human mitogenome has more than expected inverted palindromes potentially forming secondary structures after each of these transformations [45]. Further analyses show that homopolymer triplets (AAA, CCC, GGG, TTT) that promote transcriptional frameshift [46] are overrepresented in mitogenomic regions covered by detected delRNAs [47]. DelRNAs have less than expected circular code codons, codons presumably regulating transcriptional [47] and ribosomal translation frames [48–51].

Here we use del-transformed versions of the human mitochondrial genome to mimic and detect actual noncanonical RNAs in two transcriptome datasets, one originating from the whole cell and one from purified mitochondrial lines. RNAs aligning with the transformed versions of the mitogenome are considered produced by del-transcription. To study noncanonical delRNAs, we focus on the mitochondrial genome because the entire mitogenome is transcribed i.e coding and non-coding sequences on both mitogenome strands are transcribed [52], and the short mitogenome is compatible with current computational limitations for analyses that consider transformations such as systematic deletions.

**Fig 2:**
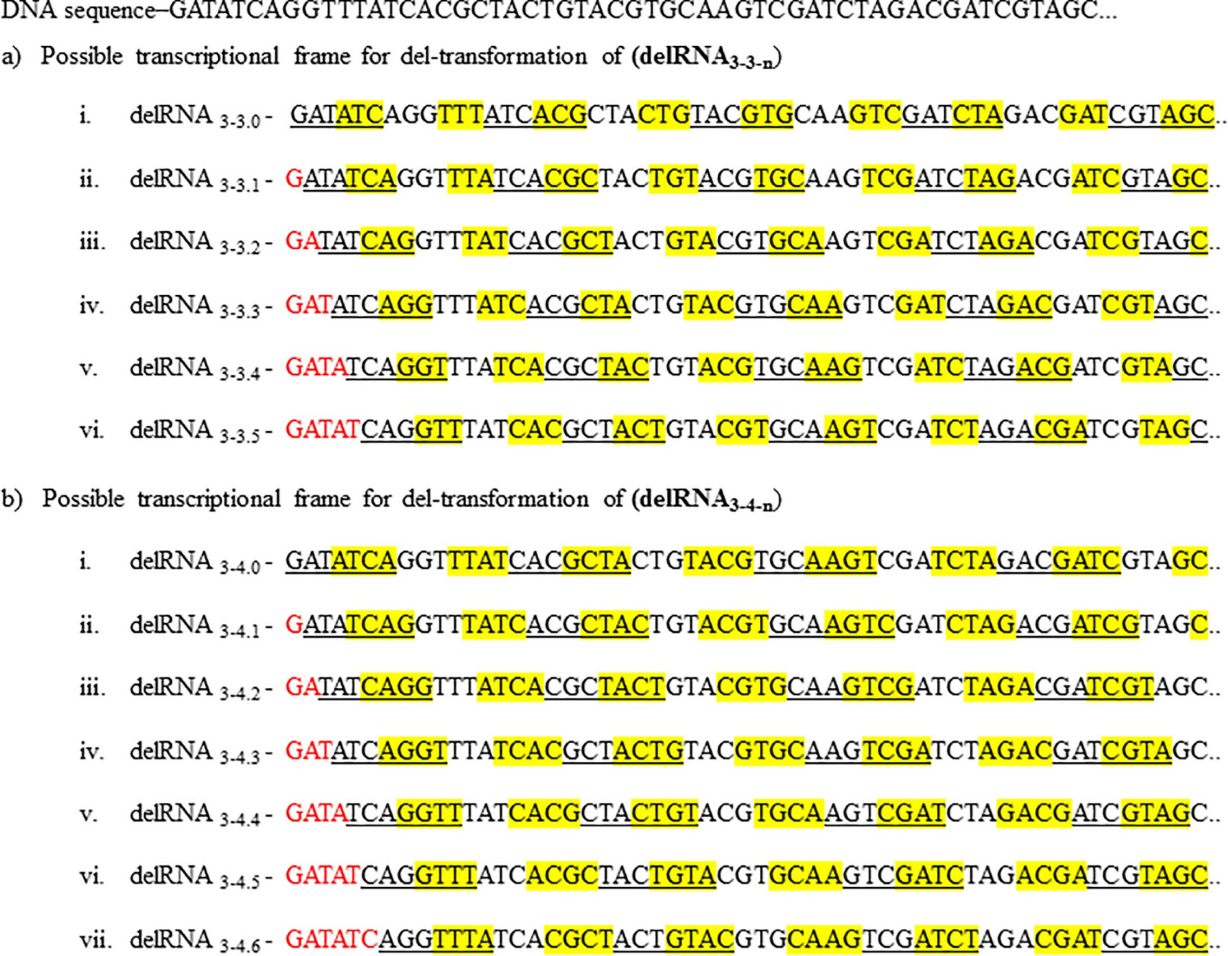
Mitogenomic versions of delRNA_3-3_ and delRNA_3-4_. Nucleotides highlighted yellow are deleted during transcription. (A) Possible mitogenomic versions for del-transcription window size six, k = 3 (delRNA_3-3.n_). (B) Possible mitogenomic versions for del-transcription window size seven, k = 4 (delRNA_3-4-n_). ‘n’: nucleotides deleted before del-transcription initiation assumed to cover all possibilities (highlighted red).

Here the principle of systematic deletions after each transcribed triplet is examined beyond deletion of mono- and dinucleotides [43], up to deletions of twelve nucleotides. Systematic deletions can start at different positions on a sequence, defining the transcriptional deletion 'frame', each producing different delRNAs. Numbers of potential deletion frames increase with deletion size (Fig 2), 3+k delRNAs for deletion size k. We also search for potential punctuation motifs signaling del-transcription, according to the previous study on homopolymers and circular code codons in delRNA_3-1_ and delRNA_3-2_ [47]. DelRNAs could result from enzyme slippage caused by homopolymers (AAA, CCC, GGG, TTT). However, on the other hand, delRNAs are expected to have less number of codons that usually conserves transcription- and/or translation frame, such as the natural circular code identified in prokaryotes and eukaryotes [48–51], a set of 20 comparatively conserved codons (AAC, AAT, ACC, ATC, ATT, CAG, CTC, CTG, GAA, GAC, GAG, GAT, GCC, GGC, GGT, GTA, GTC, GTT, TAC, TTC) [53] that enables ribosomal translation frame retrieval [48,54,55] and apparently conserves transcriptional frames [47]. Punctuations by homopolymers and circular code codons have opposite roles, therefore they are expected over-, and under-represented in detected delRNAs, respectively. These patterns have been previously found for delRNA_3-1_ and delRNA_3-2_ [47]. We expect similar patterns for k = 3 to k = 12: over- and under-representation of homopolymers and circular code codons, respectively.

Analyses of whole cell transcriptome data [56] are replicated on the transcriptome data extracted from purified mitochondrial lines [57] and on GenBank's human EST database, to test del-transcription reproducibility across independent datasets and sequencing techniques, and verify that results are not due to confounding effects from cytosolic RNAs matching del-transformations of the mitogenome by chance. To test the hypothesis of transcriptional frame retrieval, analyses of the universal circular code codons (X) identified in the genes of bacteria, eukaryotes, plasmids, and viruses [48–51] are compared with the circular code codons identified in the protein-coding genes of mitochondria (ACA, ACC, ATA, ATC, CTA, CTC, GAA, GAC, GAT, GCA, GCC, GCT, GGA, GGC, GGT, GTA, GTC, GTT, TTA, TTC) [58]. This candidate mitochondrial circular code includes 13 among 20 codons in common with the universal circular code but differs from the latter because it is not self-complementary, and its circular permutations do not produce circular codes (notated as the "C^3^" property occurring in the universal circular code). This mitochondrial circular code is derived from a much smaller sample than the universal circular code and hence could result from sampling biases. Nevertheless, if it is relevant to mitochondrial frame detection, the mitochondrial circular code should show stronger negative associations with detected delRNAs than the universal circular code.

## Methods

### *In silico* del-transformations of the human mitogenome

The human mitogenome (NC_012920.1) is transformed according to systematic deletions of 1 to 12 nucleotides after each transcribed nucleotide triplet as previously presented for deletion sizes 'k = 1' and 'k = 2' (Fig 1 in [43], Fig 1 in [59], Fig 1 in [60], Fig 2 in [47], Fig 2 in [61]). For example, to construct delRNAs with k = 3 (dRNA_3-3_) (Fig 2), after each transcribed trinucleotide, the next trinucleotide is deleted/not transcribed. The same principle produces delRNA_3-4_ (3 nucleotides transcribed followed by 4 deleted nucleotides), delRNA_3-5_ (3 nucleotides transcribed followed by 5 deleted nucleotides), delRNA_3-6_ (3 nucleotides transcribed followed by 6 nucleotide deletion) and so on until k = 12.

We produce *in silico* 3+k del-transformed versions of the human mitogenome, where each version corresponds to a different deletion frame (Fig 2). For example, a del-transformed version of the mitogenome with three transcribed nucleotides followed by deletion of the next three nucleotides (k = 3) is constructed and noted as “delRNA_3-3.0_” (Fig 2A.i). Similarly, a second mitogenome version follows the same systematic transcription/deletion pattern besides that it initially deletes the first nucleotide of the transformed sequence. This transformation is noted delRNA_3-3.1_ (Fig 2A.ii). A third mitogenome version excludes the 2 first nucleotides of the genome, then the rest of the genome is assumed transcribed along with the same systematic transcription/deletion pattern, noted delRNA_3-3.2_ (Fig 2A.iii). A fourth mitogenome version deletes the 3 first nucleotides before initiating the systematic transcription/deletion pattern delRNA_3-3.3_ (Fig 2A.iv). The fifth mitogenome version excludes the 4 first nucleotides, then applies the above systematic transcription/deletion pattern, noted delRNA_3-3.4_ (Fig 2A.v). A sixth mitogenome version excludes the 5 first nucleotides, then applies the above systematic transcription/deletion pattern, noted delRNA_3-3.5_ (Fig 2A.vi). Similarly, Fig 2B shows all possible del-transformed mitogenome versions for delRNA_3-4_ (i.e. k = 4), producing seven del-transformed versions of the mitogenome. For delRNA_3-3_, the six del-transformation frames include all possible theoretical delRNA transformations for transcription of the first three nucleotides followed by the deletion of the next nucleotide triplet (k = 3). There are 3+k del-transformed versions of any sequence. In total 114 del-transformed mitogenome versions were created along deletion sizes 1 to 12 (S1 File).

### Whole cell transcriptome analyses

Analyses below follow methods previously described for the transcriptome analyses of delRNA_3-1_ and delRNA_3-2_ [43]. We analyse seventy-one samples (SRX768406-SRX768476) [56] of human transcriptomic datasets available in the Sequence Read Archive (SRA) of GenBank. Twenty SRA entries were analysed simultaneously by BLASTN detecting RNA reads aligning with *in silico* constructed delRNA versions of the human mitogenome. We used default alignment criteria for BLASTN searches.

### Transcriptome analyses of purified mitochondrial lines

The same BLASTN analysis method was applied to transcriptome data (SRX084350-SRX084355 and SRX087285) extracted from purified mitochondrial lines [57]. BLASTN results of both whole cell and purified mitochondrial transcriptomes were compared to test the detection reproducibility of delRNA coverages.

### EST database search

The same 114 del-transformed mitochondrial sequences as used to search SRA data are used to search for ESTs matching delRNAs in GenBank's human EST database. MegaBLAST parameters (word size of 16 and gap cost (Existence: 5, Extension: 2)) were tailored to ensure inclusion of short delRNAs matching ESTs. DelRNAs detected by MegaBLAST with more than 90% identity with input del-transformed mitogenome versions were scrutinized for further analyses.

Since both transcriptome and ESTs database originate from independent studies, delRNAs detected in these datasets were mapped on the mitogenome. In the case that delRNAs are random sequencing artefacts, or random BLAST hits due to nuclear DNA/RNA contamination, overlaps between mitogenome sequences covered by del-RNAs originating from ESTs and SRA datasets should be rare and follow random predictions. Positive bias for overlapping sequences would be considered as evidence for reproducibility in delRNA detection across independent experiments and independent sequencing methods, and strong confirmations that del-transcription exists.

### Control sequence

A randomized mitogenomic sequence with the same nucleotide composition and size as the natural human mitogenome was created as a negative control. The randomized mitogenome had no significant sequence similarity with the actual mitogenome. This randomized mitogenome was used to produce 114 del-transformed (k = 1 to 12) randomized mitogenomic versions as explained in Fig 2. These sequences were separately used as a control for BLASTN and MegaBLAST searches and analyses.

However, to eliminate any false positive alignments, delRNAs that aligned in SRA/EST database and also mapped on natural mitogenome sequence (NC_012920.1). delRNAs that mapped with significant identity were removed from further analysis.

## Results and discussion

### BLASTN detects delRNAs with k = 1 to 12 in the human whole cell and purified mitochondrial transcriptomes

BLASTN yields alignment of 11869 reads (Sheet A in S2 File) with the 114 del-transformed versions of the human mitogenome, as detected within 71 publicly available human whole cell transcriptome datasets (SRX768406-SRX768476). Contig alignment of these reads gave 968 delRNA contigs (Sheet B in S2 File) with mean length of ~36bp, with average identity >90%. Similarly, 114 del-transformed versions of the human mitogenome aligned with 5084 SRA reads (Sheet A in S3 File) in 7 publicly available transcriptome datasets from purified human mitochondrial lines (SRX084350-SRX084355 and SRX087285). The mean length of 1767 detected delRNA contigs is 29.354bp, with mean average identity >93% (Sheet B in S3 File).

DelRNAs in both transcriptomic datasets were mapped on the mitogenome to find numbers of delRNAs covering the same mitogenomic regions. Details about delRNAs detected in the whole cell and purified mitochondrial transcriptome, i.e. the number of reads in BLASTN search for each delRNA, their position in the transformed mitogenome, percentage identity and the length of each delRNA for all deletion sizes are enclosed in S2 and S3 Files. A total of 367 delRNAs in the whole cell transcriptome (S2 File) and 390 delRNAs in the purified mitochondrial transcriptome (S3 File) had more than 2 reads.

Sequencing errors producing RNAs that artificially differ from the original natural sequences typically occur specifically for one specific strand, as cDNA libraries consist of double-stranded DNA produced on the template of natural RNA. DelRNAs could result from such sequencing artefacts. This possibility would be confirmed if delRNAs covering the same genome region overwhelmingly originate from the same DNA strand. On the contrary, if delRNAs originate from both strands, sequencing artefacts are far less likely, confirming that delRNAs are natural phenomena. This method has been previously used to confirm that A→I hyper-edited reads are not sequencing artifacts [29]. Indeed, 75.38% of delRNAs having more than 2 reads originate from both + and—strands (Sheet B in S2 File and Sheet B in S3 File). This result suggests that most delRNAs detected in this study are not sequencing artifacts.

### Whole cell vs purified mitochondrial transcriptomes

Surprisingly, coverages of del-transformed mitogenomes by detected delRNAs are greater for transcriptome data extracted from purified mitochondrial lines than those extracted from whole cells for 97 among 114 del-transformations of the human mitogenome (85.1%) (S4 File). This might be an effect of internal cutoffs automatically set by BLASTN considering the size of the analysed dataset (the purified mitochondrial line dataset includes fewer reads than the whole cell data). This could also reflect technical and/or biological effects specific to each experiment. Independently of this, this result shows that a wide majority of detected delRNAs are not false positives due to confounding effects of large populations of cytosolic RNAs. At most, few isolated delRNAs might be false positives due to cytosolic contaminations.

**Fig 3:**
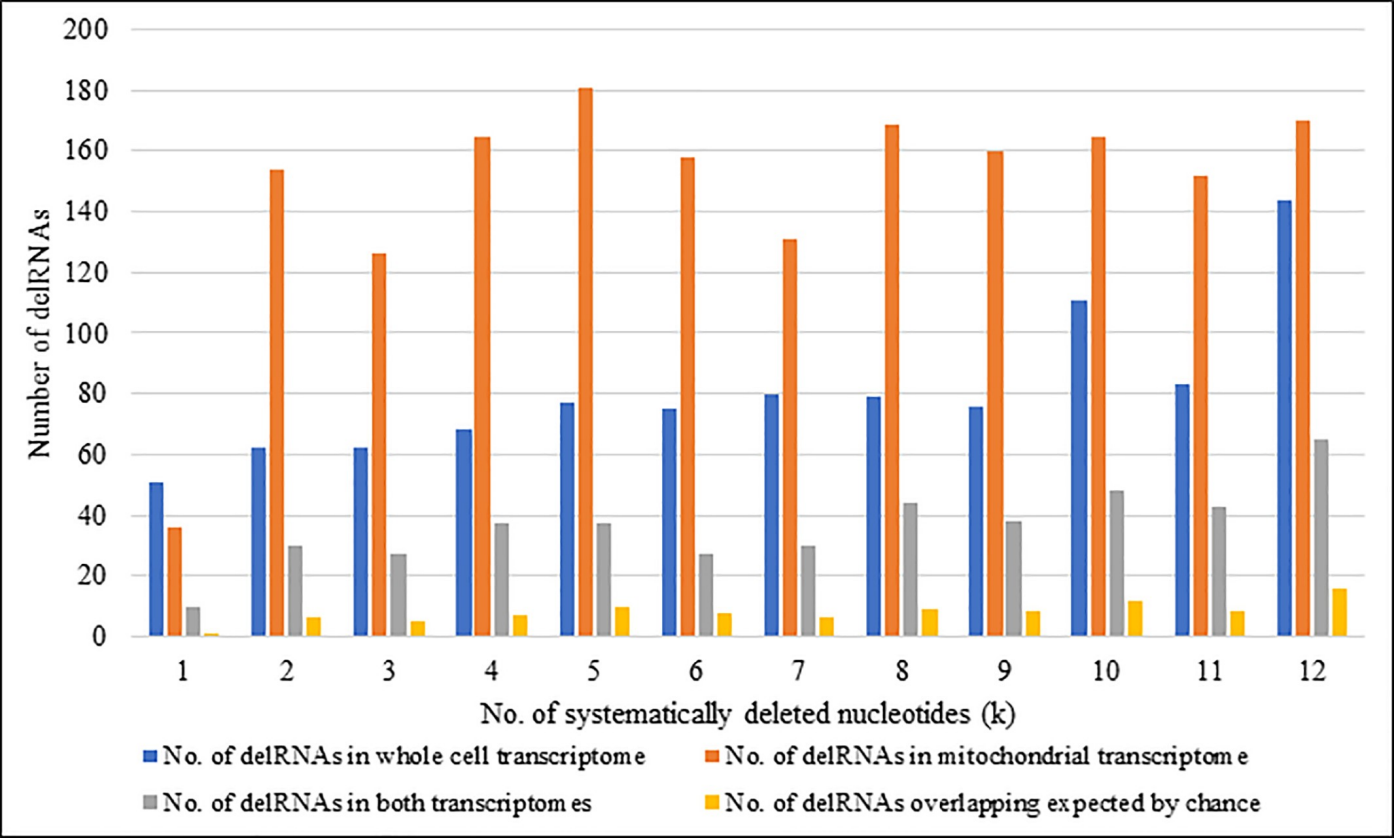
Number of delRNAs detected in whole cell and mitochondrial transcriptome for each systematic nucleotide deletions (k).

Mitogenome sequences covered by delRNAs detected in both whole cell and purified mitochondrial data are more frequent than expected by chance for 109 among 114 del-transformed versions of the mitogenome (95.6%) (S4 File). Fig 3 gives an overview of number of delRNAs detected in both datasets along with number of overlapping delRNAs in both datasets compared to number of overlaps estimated by chance for each systematic nucleotide deletion (k = 1 to 12). All 12 del-transformations (k = 1 to 12) across both transcriptomes had more overlapping delRNAs than expected by chance (P = 0.000122 according to a one-tailed sign test using the binomial distribution). Overall, 436 delRNAs detected in both transcriptomes were overlapping same mitogenomic region with average length of ~24bp. This overlapping of delRNAs is four times more frequently than expected by chance (S4 File).

Del-RNAs detected for a del-transformed randomized version of the human mitogenome in the mitochondrial transcriptome were also mapped on their respective del-transformed genome. Coverages of delRNAs detected in the purified mitochondrial transcriptome were higher for 11 among 12 deletion sizes (k) than for the randomized mitogenome sequence with the same deletion size. Obtaining this result across 12 del-transformations has P = 0.00158 (one tailed sign test, binomial distribution). Hence delRNA detections are reproducible across independent SRA transcriptome datasets and sequencing methods, and are not confounded by random matches.

### MegaBLAST detects delRNAs in EST database

The Genbank ESTs aligned with the 114 del-transformed versions of natural mitogenome and randomized del-transformed mitogenome were mapped on respective del-transformed mitogenomic version. The total mean coverage by delRNAs for each deletion size (i.e k = 1 to 12) is higher for del-transformed versions of the natural mitogenome than del-transformed versions of randomized genome (S1 Fig). Obtaining this result across all 12 deletion sizes (k) has P = 0.000122 (one tailed sign test according to the binomial distribution). Fewer randomized del-transformed mitogenome versions obtained hits with the EST database than the del-transformed versions of the natural mitogenome (one tailed P = 0.0027, Fisher exact test) (Sheet A and B in S5 File). EST-delRNAs having more than 90% identity were further considered for analyses (S6 File). In total, 1395 ESTs were detected for 111 among 114 del-transformed mitogenomic versions in Genbank's human EST database (S6 File). The mean length of these delRNAs is 24 base pairs with average identity of 97.76%. The del-transformed randomized mitogenome sequence matches with 602 ESTs across 98 among 114 del-transformed randomized version (Sheet B in S5 File). The mean length of EST-delRNAs is comparatively smaller than the delRNAs detected in mitochondrial and whole cell transcriptome. This difference in mean length of delRNAs in the EST database could be due to the stringent MegaBLAST search used for delRNAs in Genbank’s human EST database than BLASTN search used for transcriptome search. The length of ESTs aligned with delRNA_3-1_ was larger than the rest del-transformed versions (k>1) (S6 File). We believe that this difference in size of EST-delRNAs is due to the limitation of BLAST search algorithm to allow alignment of sequences with more than one gap after each trinucleotide, affecting the alignment score.

DelRNAs detected in Genbank's human EST database were mapped with the delRNAs detected in purified mitochondrial transcriptome and whole cell transcriptomic data to test for overlaps between delRNA coverages from these independent experimental datasets and sequencing methods.

#### DelRNAs in Genbank's human EST database overlapping delRNAs in purified mitochondrial transcriptome

**Fig 4:**
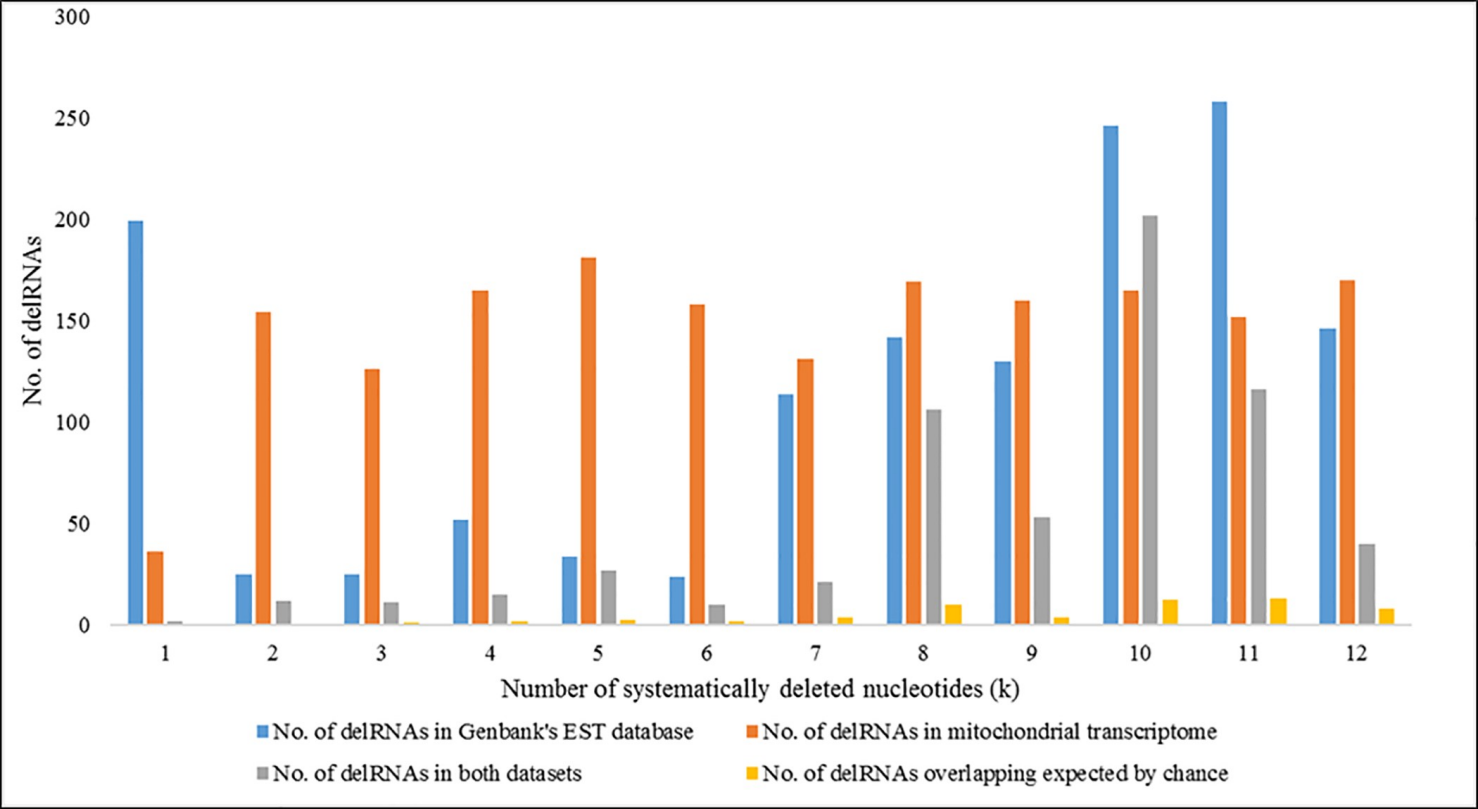
Number of delRNAs detected in Genbank’s Human EST database and purified mitochondrial transcriptome for each systematic nucleotide deletions (k).

Fig 4 shows delRNAs detected in the purified mitochondrial transcriptome, the EST database and numbers of delRNAs overlapping the same mitogenomic region and overlaps expected by chance for each systematic nucleotide deletion (k). Among a total of 1395 delRNAs from the EST database (S6 File), 615 (44.02%) EST-delRNAs overlapped with delRNAs detected in the mitochondrial transcriptome. Overall, these overlaps are 8 times more frequent than expected by chance (Fig 4, S7 File). DelRNAs in the EST database were detected in 105 del-transformed mitogenomic versions, among which 72 (63.15%) del-transformed versions had at least one overlapping delRNA with the delRNAs detected in the mitochondrial transcriptome. Among those 72 del-transformed versions, 71 (62.28%) had more delRNA overlaps than expected by chance with average overlap length of 22 nucleotides (S7 File).

#### DelRNAs in Genbank's human EST database overlapping delRNAs from the whole cell transcriptome dataset

**Fig 5:**
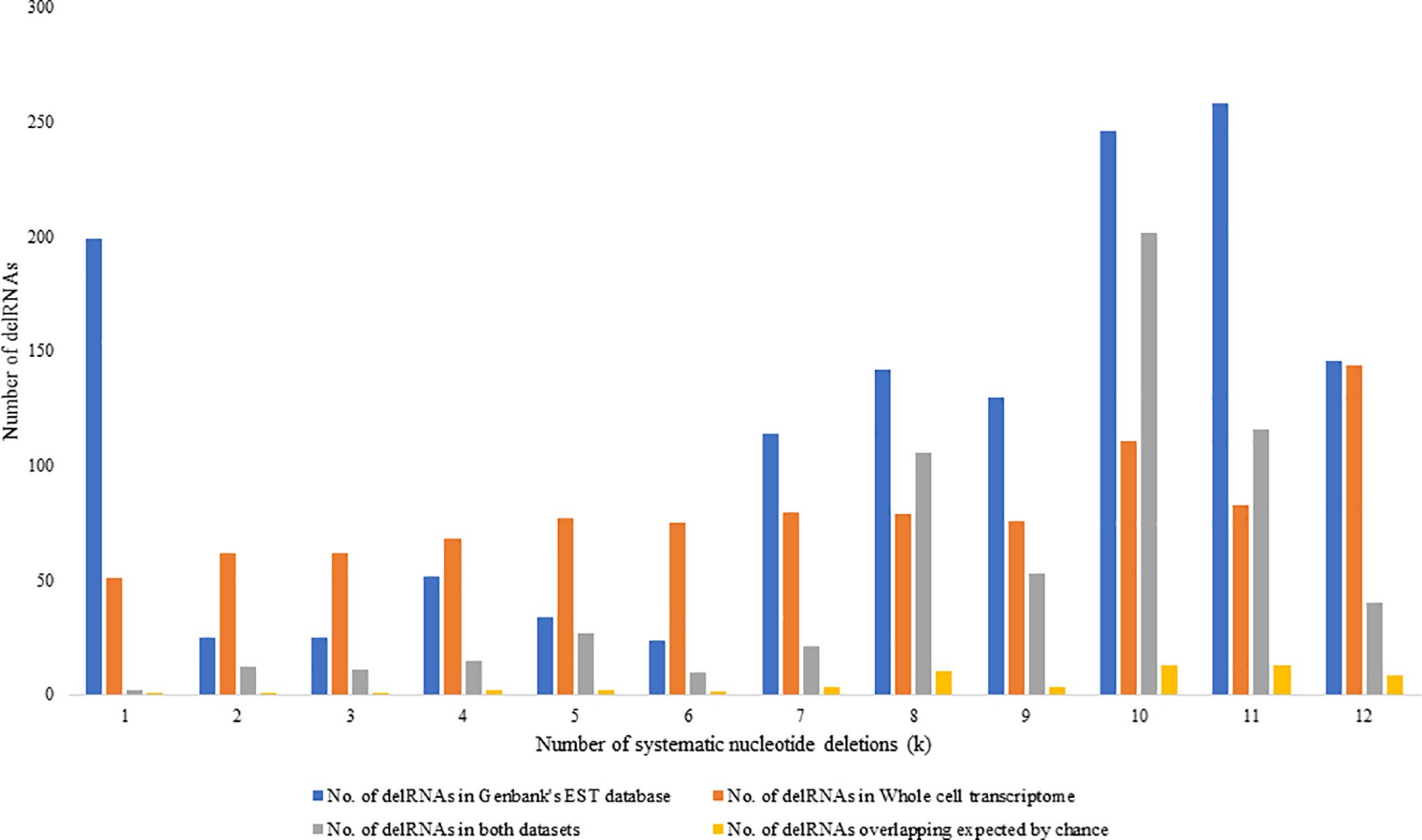
Number of delRNAs detected in Genbank's human EST database and whole cell transcriptome transcriptome for each systematic nucleotide deletions (k).

Fig 5 shows numbers of EST-delRNAs overlapping with delRNAs detected in the whole cell transcriptome database for each systematic nucleotide deletion (k). A total of 388 delRNAs in whole cell transcriptome overlapped 1395 (27.81%) EST-delRNAs with average overlap of 23 nucleotides (S8 File). Overlapping delRNAs were observed in 63 among 114 (55%) del-transformed versions of the mitogenome among which 61 (53.51%) del-transformed mitogenome versions had more overlaps than expected by chance.

**Fig 6:**
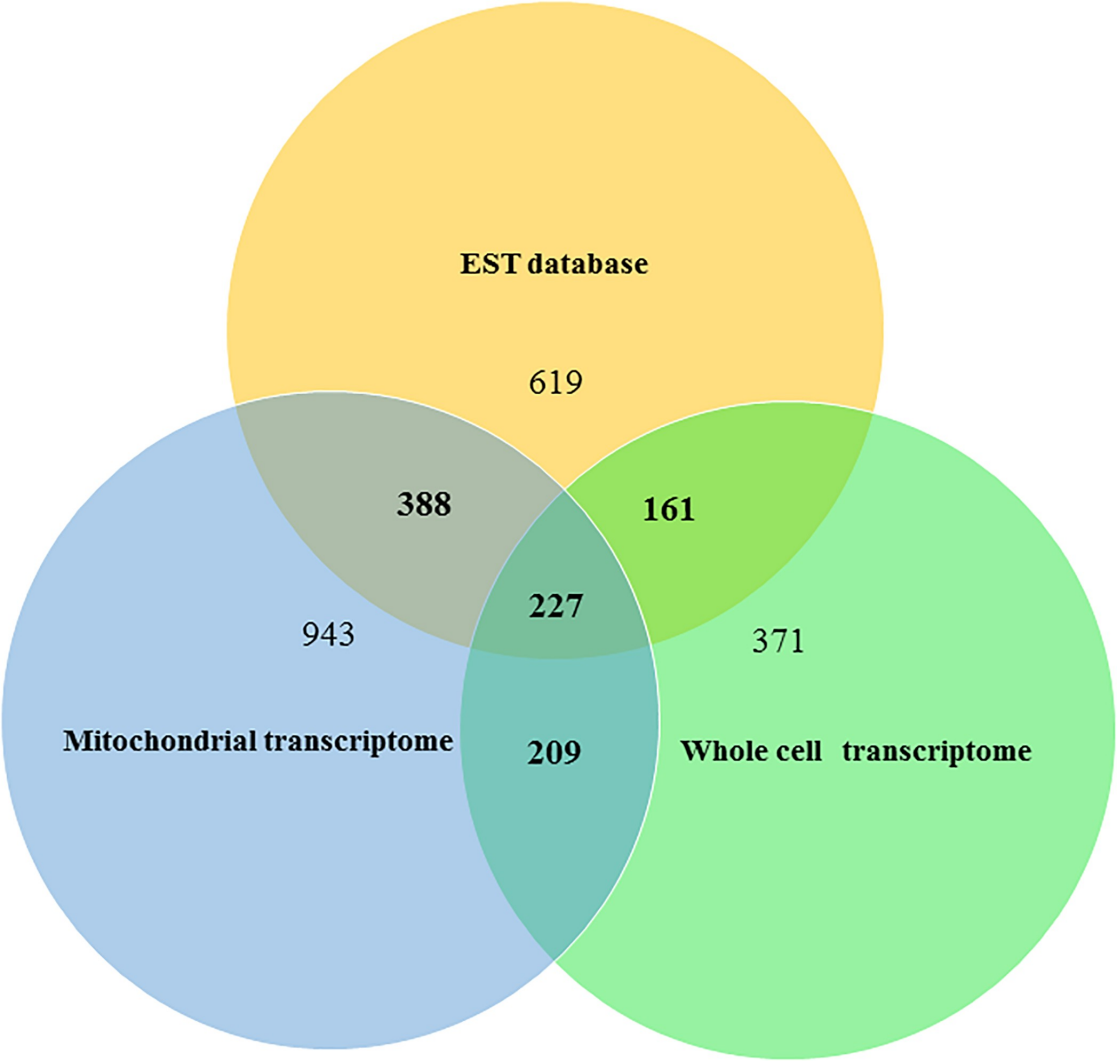
Numbers of overlapping delRNAs in three different datasets. The Venn diagram shows the number of overlapping delRNAs across the three independent datasets and numbers of delRNAs not overlapping with any of the other two datasets. Total numbers of delRNAs detected in the whole cell transcriptome, the mitochondrial transcriptome and the human EST datasets are 968, 1767 and 1395, respectively.

Fig 6 gives an overall view of the delRNAs detected in all three datasets. Among 1395 delRNAs detected in Genbank's EST database, 777 (55.70%) EST-delRNAs overlap with delRNAs detected in either whole cell and/or mitochondrial transcriptomes. Among 436 delRNAs detected in both transcriptomes, 227 delRNAs overlapped EST-delRNAs (S9 File), confirming that there are real short delRNAs non-canonically transcribed from the human mitogenome. Overlapping delRNAs in all three independent datasets are strong evidence that deletion transcription is a true phenomenon and not a sequencing artefact or contamination. Analyses below also underline the mitogenomic regions that are hotspots for deletion-transcriptions.

#### Association of delRNAs with protein coding genes

**Fig 7:**
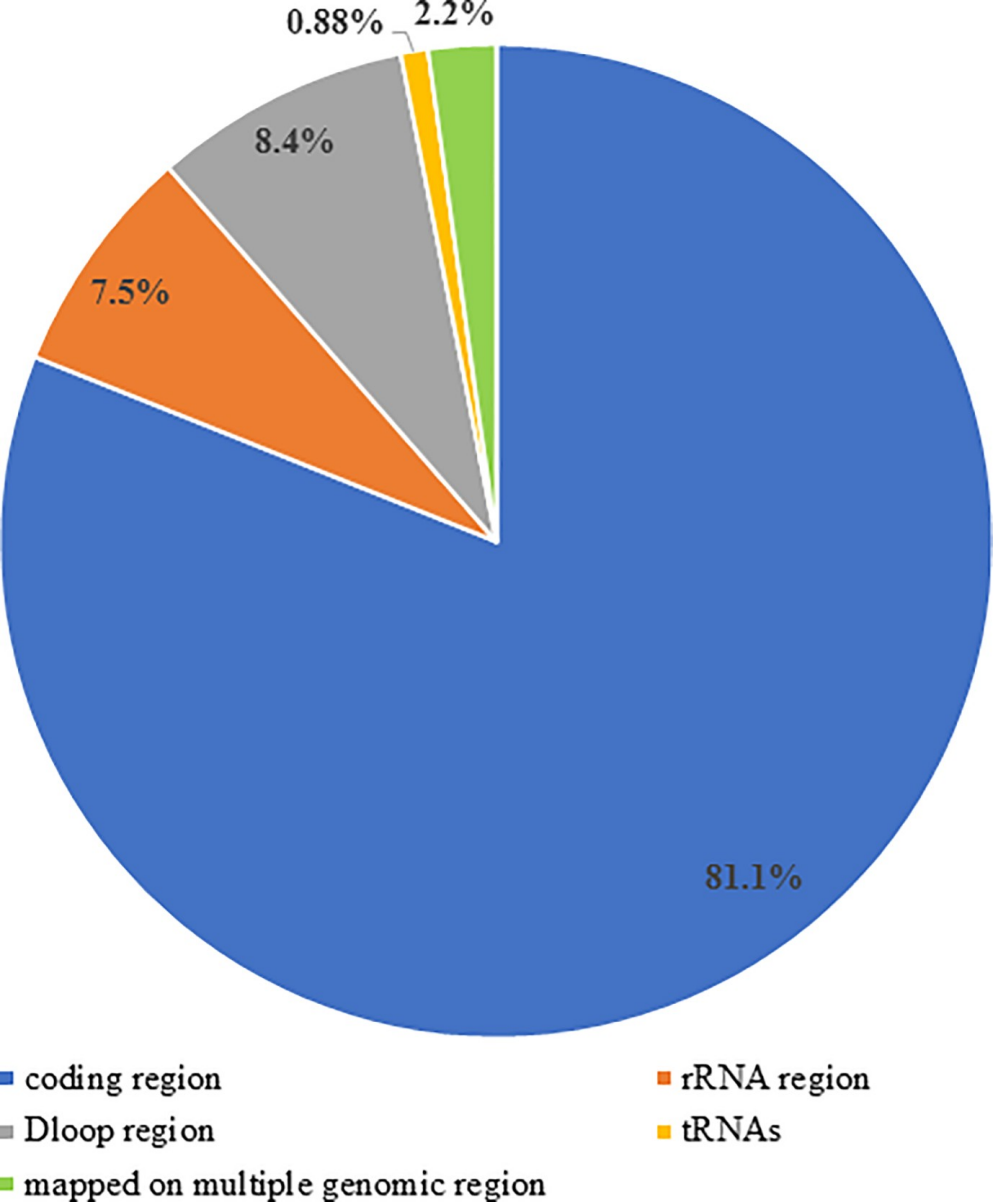
DelRNAs detected in three independent datasets mapped on coding and dloop hypervariable region. Mitogenome composition (Protein coding genes: 68.44%, Dloop: 6.7%, rRNA region: 15.17%, tRNA region:9.35%).

The 227 EST-delRNAs (S9 File) that were also detected in whole cell and purified mitochondrial transcriptome were mapped on the mitochondrial genome (NC_012920.1) to determine if these del-transformation is associated with coding or non-coding region. However, with the increase in number of systematic deletions (k), the coverage of delRNAs will increase. Therefore, it was expected that most of these delRNAs will overlap on both coding and non-coding region making it difficult to link these delRNAs to any particular mitogenomic region. Surprisingly, among 227 EST-delRNAs, 222 (97.8%) mapped on either coding or noncoding region. Fig 7 gives an overview of number of delRNAs mapped on mitogenomic regions. Among 227 EST-delRNAs, 184 (81.05%) mapped on protein coding genes, 17 (7.5%) mapped on rRNA region, 19 (8.4%) on dloop, 2 (0.88%) and 5 delRNAs mapped on more than one mitogenomic regions. Interestingly, all the 19 delRNAs of dloop mapped on the hypervariable region 2 and 3 (HVR2 and HVR3; coverage 3.47% of mitogenome) suggesting possible association of delRNAs with HVR2. Results indicate that del-transformation is rare in conserved tRNA region and rRNA region whereas it is associated with dloop-hypervariable and protein coding regions of mitogenome. Since with increase in systematic deletion size the coverage of delRNAs on natural mitogenome increases. Therefore, we could not find an association among delRNAs and disease-causing mutations. DelRNAs that mapped on dloop hypervariable region had homopolymer frequency 2.85 times than the remaining del-transformed versions (S9 File). Similarly, 184 delRNAs mapping on protein coding genes had an overall homopolymer frequency 1.98 times than remaining del transformed mitogenome.

#### Homopolymers in delRNAs

Homopolymer nucleotide triplets (AAA, CCC, GGG, TTT) cause frameshifts during DNA replication and transcription [46,62] and ribosomal slippages during translation [46,63–66]. Frequencies of these homopolymers in detected delRNAs are compared with their frequencies in the remaining transformed mitogenome not covered by detected delRNAs. Table 1 shows frequencies of homopolymers in detected delRNAs for each del-transformation (k = 1 to k = 12) in the whole cell and purified mitochondrial line transcriptomes. In both datasets, across all del-transformation (k = 1 to k = 12), frequencies of homopolymers in detected delRNAs is higher than homopolymer frequencies in the remaining del-transformed mitogenome (Table 1). Obtaining this result across all 12 del-transformations has P = 0.000122 according to a one-tailed sign test using the binomial distribution. Chi-square tests show that the difference in homopolymer frequencies between regions covered by delRNAs and those not covered by delRNAs is statistically significant at P <0.05 for each del-transformation, for each whole cell and purified mitochondrial transcriptomes. The only exception is for delRNA_3-6_ for the whole cell transcriptome data (Table 1).

**Table 1:** Homopolymer frequencies of delRNAs detected in whole cell and purified mitochondrial transcriptome.

	delRNA_3-k_	No. of trinucleotides		No. of homopoly-mers in delRNAs	Percent Homopolymers (%)		Ratio (A/B)	P of Chi-square
		In detected delRNAs	In remaining del-transformed mitogenome		In detected delRNAs (A)	In remaining del-transformed mitogenome (B)		
Whole cell transcriptomes	delRNA_3-1_	607	15962	76	12.5	8.74	1.43	0.00
	delRNA_3-2_	723	15846	116	16.04	8.55	1.87	0.00
	delRNA_3-3_	766	15803	90	11.77	8.74	1.34	0.00
	delRNA_3-4_	879	15690	125	14.22	8.58	1.66	0.00
	delRNA_3-5_	1078	15491	136	12.62	8.62	1.46	0.00
	delRNA_3-6_	911	15658	92	10.1	8.81	1.15	0.18
	delRNA_3-7_	963	15607	113	11.73	8.7	1.35	0.00
	delRNA_3-8_	1019	15555	131	12.86	8.61	1.49	0.00
	delRNA_3-9_	980	15589	143	14.59	8.52	1.71	0.00
	delRNA_3-10_	1386	15189	181	13.06	8.49	1.54	0.00
	delRNA_3-11_	1079	15497	137	12.7	8.61	1.47	0.00
	delRNA_3-12_	1846	14729	233	12.62	8.41	1.5	0.00
Purified mitochondrial cell line transcriptomes	delRNA_3-1_	358	16211	43	12.01	8.81	1.36	0.02
	delRNA_3-2_	1581	14988	201	12.71	8.47	1.50	0.00
	delRNA_3-3_	1309	15260	168	12.83	8.54	1.50	0.00
	delRNA_3-4_	1694	14875	201	11.87	8.54	1.39	0.00
	delRNA_3-5_	1885	14684	191	10.13	8.72	1.16	0.02
	delRNA_3-6_	1612	14957	193	11.97	8.55	1.40	0.00
	delRNA_3-7_	1395	15174	156	11.18	8.66	1.29	0.00
	delRNA_3-8_	1812	14757	217	11.98	7.6	1.58	0.00
	delRNA_3-9_	1624	14945	217	13.36	8.39	1.59	0.00
	delRNA_3-10_	1400	15169	180	12.86	8.51	1.51	0.00
	delRNA_3-11_	1240	15329	213	17.18	8.21	2.09	0.00
	delRNA_3-12_	1317	15252	213	16.17	8.25	1.96	0.00
Mitogenomic regions covered by delRNAs in whole cell and purified mt RNA data	delRNA_3-1_	108	16461	14	12.96	8.85	1.46	0.09
	delRNA_3-2_	240	16329	47	19.58	8.72	2.24	0.00
	delRNA_3-3_	266	16303	42	15.79	8.76	1.80	0.00
	delRNA_3-4_	327	16242	47	14.37	8.77	1.64	0.00
	delRNA_3-5_	370	16199	55	14.86	8.74	1.70	0.00
	delRNA_3-6_	230	16339	28	12.17	8.88	1.38	0.06
	delRNA_3-7_	269	16300	37	13.75	8.80	1.56	0.00
	delRNA_3-8_	403	16166	57	14.14	7.87	1.80	0.00
	delRNA_3-9_	320	16249	59	18.44	8.69	2.12	0.00
	delRNA_3-10_	369	16200	65	17.62	8.68	2.03	0.00
	delRNA_3-11_	272	16297	68	25.00	8.61	2.90	0.00
	delRNA_3-12_	436	16133	78	17.89	8.63	2.07	0.00

The table shows the percentage of homopolymers in delRNAs detected for each del-transformation in whole cell and purified mitochondrial cell line transcriptomes. It also shows the homopolymer percentage in mitogenomic regions covered by overlapping delRNAs detected in both whole cell and purified mitochondrial RNA datasets. Homopolymer frequencies in detected delRNAs are higher for all del transformations in the whole cell transcriptome, mitochondrial transcriptome and mitogenomic regions covered by delRNAs from both RNA datasets than in the remaining del-transformed mitogenome. Considering the frequencies of A, C, G and T in the human mitogenome, the expected frequency of homopolymer triplets is 0.077496. All results find more homopolymer triplets in del-transformed human mitogenome versions than expected using the mononucleotide frequencies: A = 0.303931257 C = 0.31269238, G = 0.13090712 and T = 0.24708794. This is also the case for the untransformed human mitogenome, for which the expected homopolymer triplet frequencies are AAA = 0.02959325; CCC = 0.03057397; GGG = 0.00224331 and TTT = 0.01508532 (total 0.07749586) and the observed frequency is 0.0887199.

Overall, homopolymer frequencies were 1.5X times higher in detected delRNAs than in the remaining del-transformed mitochondrial sequences for analyses of both transcriptome datasets (Table 1). This shows that polymerase slippage at homopolymers contributes to non-canonical del-transcription. These patterns are incompatible with spurious detections of delRNAs by alignment searches among massive sequence data.

To further strengthen our findings, we scrutinized homopolymer frequencies in overlapping delRNAs detected independently in the transcriptomes of the whole cell and purified mitochondrial cell lines. Among 12 del-transformations (k = 1 to 12), the percentage of homopolymers in overlapping delRNAs is greater than in delRNAs detected only once, either in the whole cell or the purified mitochondrial cell line transcriptomes in 11 among 12 comparisons (Table 1). This result has P = 0.00158 according to a one-tailed sign test using the binomial distribution.

Homopolymer frequencies of delRNAs detected in Genbank’s human EST database unsurprisingly gives similar result. DelRNAs detected for all 12 systematic nucleotide deletion (k) have overall higher homopolymer frequencies (P = 0.000122 according to a one-tailed sign test using the binomial distribution). Similarly, homopolymer frequencies of EST-delRNAs mapping with delRNAs detected in whole cell and mitochondrial transcriptome (S10 File) are higher for all delRNAs from k 1 to 12 with P = 0.000122 as per one-tailed sign tests using the binomial distribution, respectively.

Detection of overlapping delRNAs from two independent transcriptomes and in Genbank’s human EST database in itself supports our hypothesis of del-transcription, confirming result reproducibility. The higher homopolymer frequencies in overlapping delRNAs, as compared to those detected only in one dataset, indicates that high homopolymer densities increase the frequency of del-transcription, and further confirms that del-transcription exists.

#### Circular code and delRNAs

The natural circular code (X) is a specific set of 20 codons universally overrepresented in the coding frame of protein-coding genes as compared to their remaining, non-coding frames [48]. As a group, codons of X enable recognizing the coding frame [55,67,68]. Though mechanisms by which this occurs are still unknown, it is believed that stretches of codons belonging to X enable ribosomes to recognize the translation frame [48,68–70]. Distributions of triplets belonging to X in the structures formed by ribosomes [70] and tRNAs [69,71] suggest that X motifs in these molecules central to translation play roles in recognizing protein-coding frames. Previous analyses of delRNA_3-1_ and delRNA_3-2_ showed that X also regulates the frame of deletion transcriptions [47].

Here frequencies of motifs corresponding to the universal circular code X proposed for the protein-coding genes in prokaryotes and eukaryotes [48,50,51] in detected delRNAs (considering the nucleotide triplets that are not separated by deletions) are lower in 9 among 12 del-transformations than in the rest of the mitogenome not covered by delRNAs for whole cell transcriptomic and purified mitochondrial line transcriptomic data both (Table 2). This tendency for the avoidance of the universal circular code in mitochondrial delRNAs detected in the whole cell and mitochondrial transcriptomes has P = 0.0365, respectively (one-tailed sign tests using the binomial distribution, Table 2) and a combined P = 0.004 using Fisher's method for combining P values [72], based on a chi square distribution with 2xk degrees of freedom, where k is the number of independent P values that are combined.

**Table 2:** Frequencies of codons belonging to the universal circular code X {AAC, AAT, ACC, ATC, ATT, CAG, CTC, CTG, GAA, GAC, GAG, GAT, GCC, GGC, GGT, GTA, GTC, GTT, TAC, TTC} in delRNAs detected in transcriptomic data.

delRNA_3-k_	Whole cell				Mito-transcriptome				Overlapping delRNAs	
	Percentage of X (%)		Ratio (A/B)	P of Chi-square	Percentage of X (%)		Ratio (C/D)	P of Chi-square	No. of X) codons in del-RNAs	Percentage of X (%) in del-RNAs
	In del-RNAs (A)	In remaining del-transformed mitogenome (B)			In del-RNAs (C)	In remaining del-transformed mitogenome (D)				
delRNA_3-1_	30.48	30.470	1.00	1.00	30.17	30.48	0.99	0.89	34	31.48
delRNA_3-2_	28.77	30.55	0.94	0.31	28.97	30.63	0.95	0.17	62	25.83
delRNA_3-3_	27.73	30.62	0.91.	0.006	30.10	30.50	0.99	0.76	75	28.20
delRNA_3-4_	26.73	30.68	0.87	0.01	28.39	30.71	0.92	0.05	88	26.91
delRNA_3-5_	28.85	30.59	0.94	0.23	30.13	30.52	0.99	0.73	95	25.67
delRNA_3-6_	32.49	30.36	1.07	0.17	25.25	30.86	0.82	0.00	58	25.22
delRNA_3-7_	30.22	30.49	0.99	0.86	29.89	30.53	0.98	0.62	80	29.74
delRNA_3-8_	30.62	30.45	1.01	0.92	29.80	30.55	0.96	0.51	128	31.76
delRNA_3-9_	25.92	30.76	0.84	0.00	29.67	30.56	0.97	0.47	93	29.06
delRNA_3-10_	28.93	30.60	0.95	0.19	34.21	30.13	1.14	0.00	118	31.97
delRNA_3-11_	29.38	30.54	0.96	0.42	36.69	29.97	1.22	0.00	113	41.54
delRNA_3-12_	27.20	30.87	0.88	0.00	37.05	29.90	1.24	0.00	152	34.86

Columns show their percentages in detected delRNAs and in the remaining transformed mitogenome for each del-transformed version, and the chi-square P value testing for difference in circular codon frequencies (Table 1). The last columns show their percentages in delRNAs detected in purified mitochondrial transcriptomes overlapping the same mitogenomic regions and their percentage ratio. Note: Total number of trinucleotides in the delRNAs and in remaining del-transformed mitogenome for whole cell transcriptome, mito-transcriptome and in overlapping delRNAs is given in Table 1.

We also analysed frequencies of codons belonging to the proposed mitochondrial circular code X_0_(MIT) [58]. Among 12 del-transformations, 11 del-transformations in the whole cell, and 9 del-transformations in the purified mitochondrial transcriptome (Table 3) had lower frequencies of X_0_(MIT) in detected delRNAs with P values of 0.00158 and 0.0365, respectively (one-tailed sign test using the binomial distribution for each transcriptome dataset). The combined P values according to Fisher’s method to combine P values yields P = 0.00028.

**Table 3:** Frequencies of codons belonging to the mitochondrial circular code X_0_(MIT) in delRNAs and in the remaining deletion-transformed mitogenome.

delRNA_3-k_	Whole cell transcriptome				Mito-transcriptome				Overlapping delRNAs	
	Percentage of X_0_ (MIT) (%)		Ratio (A/B)	P of Chi-square	Percentage of X_0_ (MIT) (%)		Ratio (C/D)	P of Chi-square	No. of X_0_(MIT) codons in del-RNAs	Percentage of X_0_(MIT) (%) in del-RNAs
	In del-RNAs (A)	In remaining del-transformed mitogenome (B)			In del-RNAs (C)	In remaining del-transformed mitogenome (D)				
delRNA_3-1_	29.49	31.07	0.95	0.38	30.73	31.02	0.99	0.92	32	29.63
delRNA_3-2_	28.77	31.11	0.93	0.16	27.70	31.36	0.88	0.00	57	23.75
delRNA_3-3_	25.75	30.73	0.83	0.002	26.67	31.38	0.85	0.00	70	26.31
delRNA_3-4_	29.47	31.10	0.95	0.31	29.52	31.18	0.95	0.16	94	28.75
delRNA_3-5_	27.37	31.26	0.88	0.01	28.80	31.29	0.92	0.03	100	27.03
delRNA_3-6_	30.73	31.03	0.99	0.86	25.56	31.44	0.81	0.00	50	21.46
delRNA_3-7_	29.80	31.08	0.96	0.40	29.61	31.14	0.95	0.25	82	30.48
delRNA_3-8_	27.77	31.12	0.89	0.02	28.59	31.31	0.91	0.02	106	26.30
delRNA_3-9_	27.35	31.24	0.88	0.01	29.80	31.14	0.96	0.27	92	28.75
delRNA_3-10_	29.87	31.11	0.96	0.34	35.43	30.60	1.16	0.00	112	30.35
delRNA_3-11_	31.33	30.99	1.01	0.82	36.05	30.60	1.18	0.00	114	41.91
delRNA_3-12_	28.60	31.31	0.91	0.02	36.14	30.57	1.18	0.00	175	40.13

The 20 codons belonging to the suspected mitochondrial circular code are {ACA, ACC, ATA, ATC, CTA, CTC, GAA, GAC, GAT, GCA, GCC, GCT, GGA, GGC, GGT, GTA, GTC, GTT, TTA, TTC}. Columns and description as in Table 2.

Frequencies of codons belonging to the universal circular code were also calculated for delRNAs detected in the human EST database (S11 File). Among 12 del-transformations, 6 del-transformations had higher percentages of universal X than in the remaining del-transformed mitogenome (P = 0.3063 one-tailed sign test using the binomial distribution). Eight among 12 del-transformations had higher percentages of universal X in EST-delRNAs overlapping delRNAs detected in whole cell transcriptome than for the remaining non-overlapping delRNAs detected in only a single database (P = 0.0969). Similarly, percentages of codons belonging to the circular code in EST-delRNAs overlapping delRNAs from the mito-transcriptome is higher for 9 among 12 del-transformed versions (P = 0.0365, one-tailed sign test, binomial distribution). The combined P values according to Fisher’s method to combine P values yields P = 0.0126.

Proposed mitochondrial circular code X_0_(MIT) are slightly stronger than the universal circular code for delRNAs detected in EST-delRNAs. Among 12 del-transformations, 7 del-transformations in EST database, 8 del-transformations for EST-delRNAs overlapping delRNAs detected in the whole cell transcriptome and 8 del-transformations for EST-delRNAs overlapping delRNAs detected in the mito-transcriptome had higher percentages of X X_0_(MIT) (S12 File), with P = 0.0969 according to one-tailed sign tests using the binomial distribution. Fisher’s method to combine P values yields combined P = 0.011.

Positive results were stronger for avoidance of the proposed mitochondrial circular code in delRNAs as compared to the universal circular code X identified for prokaryote and eukaryote protein-coding genes (Tables 2 and 3). These results cautiously support that a different circular code exists in mitochondria [58]. We have no explanation for the higher frequency of X (Table 2, Table 3, S11 File, S12 File) in delRNAs detected in the mitochondrial transcriptome for high k (delRNA_3-10_, delRNA_3-11_ and delRNA_3-12_).

## General discussion and future prospects

In this study we tested a phenomenon of systematic nucleotide deletion during transcription in three independent datasets. Results confirm the working hypotheses that sometimes, transcription systematically deletes nucleotides. Analyses detect delRNAs corresponding to systematic deletion of 1 to 12 nucleotides after every transcribed tri-nucleotide in the human mitochondrial transcriptome, for three independent transcriptome datasets, two sequenced by NGS methodologies and one by Sanger methodology. Numerous delRNAs were detected in all three datasets, indicating high reproducibility of the human mitochondrial del-transcriptome. DelRNAs detected in this study are around 25–30 bp long. BLASTN search of delRNA_3-1_ transformed mitogenome aligned with much longer ESTs having a systematic single gap in subject sequence and having continuous alignment at the terminals of the subject sequence (Not shown here). We believe these delRNAs could be part of much longer chimeric RNAs due to the RNA polymerase switching from canonical transcription to non-canonical del-transcription.

Homopolymers AAA, CCC, GGG and TTT, which cause polymerase slippage [46] and transcriptional frameshift [46] have higher frequencies in detected delRNAs than in the rest of the mitogenome. This suggests that these nucleotide triplets signal del-transcription. Their function seems opposite to codons belonging to the circular codes which apparently prevent del-transcription. DelRNAs might result from systematic deletions occurring during transcription, but a second possibility, RNA editing by an elusive mitochondrial spliceosome could also produce delRNAs, resembling observations from eukaryotic cytosols [73]. Circular code codons (X) enable recognizing the coding frame [55,67,68] and transcriptional frame [47]. Higher circular code frequencies in higher del-versions (k>12) of delRNAs might indicate the role of circular codes in post transcriptional editing by the mitochondrial splicesome.

DelRNAs detected in the purified mitochondrial line transcriptome have particularly high homopolymer frequencies (Table 2). Notably, detected delRNAs are poor in codons belonging to the hypothetical mitochondrial circular code, strengthening the still weak evidence indicating the existence of a different circular code in mitochondria as opposed to the universal circular code. This observation strengthens the discussion about mitochondria representing an independent branch of the tree of life [74], perhaps together with giant viruses [75].

Observations here support the hypothesis that mitochondrial genomes have greater coding potential than believed, also supported by the observations of two lncRNAs in human mitochondrial DNA [76]. An association between detected delRNAs (k = 1 and 2) and peptides matching human proteome data [43] suggest translation of delRNAs. We recommend similar mass spectrometry human proteome analyses for peptides translated from delRNAs with k = 3 to 12. Noncoding RNAs associated with ribosomes are translated into peptides [77], suggesting possible dual roles of delRNAs. Further in-depth analyses of delRNAs may help to explain various missing/hidden genes called “Dark DNA”, whose translational products are detected in mass spectrometry data [12].

## Supporting information

S1 Fig Percentage coverage by delRNAs detected in human EST database for del-transformed mitogenomes and del-transformed randomized sequences. (TIF)

S1 File Del-transformed 114 mitogenomic versions assuming k = 1 to 12 systematic nucleotide deletion. (TXT)

S2 File BLASTN search results for 114 del-transformed mitogenome in whole cell transcriptome. ‘n’ is nucleotides skipped before del-transcription starts. For delRNA_3-1.n_, ‘n’ corresponds to delRNA_3-1.0_, delRNA_3-1.1_, delRNA_3-1.2_, and delRNA_3-1.3_. 5’ and 3’ are the position of delRNAs on the transformed mitochondrial region detected in the whole cell transcriptome. Sheet2: shows the number of reads detected for each delRNA, percent identical nucleotides in the alignment (Id), the length of each delRNA, and the strand of reads detected.
(XLSX)

S3 File BLASTN search results for 114 del-transformed mitogenome in purified mitochondrial transcriptome. Description same as S2 File.
(XLSX)

S4 File Number of delRNAs detected in human whole cell and purified mitochondrial transcriptomes by BLASTN for each del-transformed mitogenomic version. Columns are: 1. systematic deletion lengths (k = 1 to 12 deleted nucleotides); 2. ‘n’: nucleotides deleted before del-transcription starts; 3–5. Numbers of delRNAs detected with their coverage on del-transformed mitogenome and average lengths for each del-transformation in whole cell transcripome data (SRX768406-SRX768476); 6–8. Numbers of delRNAs detected, coverage on del-transformed mitogenome and their average lengths in purified mitochondrial transcriptome data (SRX084350-SRX084355 and SRX087285); 9 and 10. Number of delRNAs with their average lengths detected in both whole cell and purified mitochondrial transcriptomes and covering the same mitogenomic regions; and 11. Numbers of delRNAs covering the same mitogenomic region expected by chance.
(XLSX)

S5 File MegaBLAST search results for 114 del-transformed mitogenome in Genbank’s human EST database. Sheet2: MegaBLAST results for randomized mitogenomic versions.
(XLSX)

S6 File DelRNAs detected in Genbank’s human EST database. The EST-delRNAs mapping with delRNAs detected in the whole cell transcriptome are highlighted green, and EST-delRNAs mapping with delRNAs detected in the purified mito-transcriptome are highlighted yellow. EST-delRNAs mapping along with delRNAs of both transcriptomes are highlighted blue.
(XLSX)

S7 File Number of delRNAs detected in Genbank's human EST database and in the purified mitochondrial transcriptome. Columns are: 1. systematic deletion lengths (k = 1 to 12 deleted nucleotides); 2. ‘n’: nucleotides deleted before del-transcription starts; 3–5. Numbers of delRNAs detected, coverage on del -transformed mitogenome with their average lengths for each del-transformation in the human EST database; 6–8. Numbers of delRNAs detected, coverage and their average lengths in the purified mitochondrial transcriptome data (SRX084350-SRX084355 and SRX087285); 9–10. Numbers of overlapping delRNAs overlap average lengths; and 11. Numbers of overlapping delRNAs expected by chance.
(XLSX)

S8 File DelRNAs detected in Genbank's human EST database and in the human whole cell mitochondrial transcriptome. Description as in S4 and S7 Files.
(XLSX)

S9 File EST-delRNAs detected in both transcriptome and Genbank’s human EST database along with actual position (column 14–15) and genomic region of mapping on natural reference human mitogenome. (XLSX)

S10 File Homopolymer frequencies of delRNAs detected in Genbank’s Human EST database. The table shows the percentage of homopolymers in delRNAs detected for each del-transformation in Genbank’s Human EST database. It also shows the homopolymer percentage in mitogenomic regions covered by overlapping delRNAs detected in the EST database and the whole cell transcriptome, and delRNAs detected in the EST database and the purified mitochondrial transcriptome.
(XLSX)

S11 File Frequencies of codons belonging to the universal circular code X {AAC, AAT, ACC, ATC, ATT, CAG, CTC, CTG, GAA, GAC, GAG, GAT, GCC, GGC, GGT, GTA, GTC, GTT, TAC, TTC} in delRNAs detected in Genbank’s EST database. Columns show their percentage in the delRNAs detected in Genbank’s human EST database and in the remaining transformed mitogenome for each del-transformed version, and the chi-square P value testing for difference in circular codon frequencies. The last two columns show the percentages of X in EST-delRNAs overlapping delRNAs detected in the whole cell, and in the purified mitochondrial transcriptome. Note: Total number of trinucleotides in the delRNAs and in the remaining del-transformed mitogenome for whole cell transcriptome, mito-transcriptome and in overlapping delRNAs is given in S10 File.
(XLSX)

S12 File Frequencies of codons belonging to the proposed mitochondrial circular code X_0_(MIT) {ACA, ACC, ATA, ATC, CTA, CTC, GAA, GAC, GAT, GCA, GCC, GCT, GGA, GGC, GGT, GTA, GTC, GTT, TTA, TTC} in delRNAs detected in Genbank’s EST database. Description and columns as for S11 File.
(XLSX)

## References

[1] Seligmann H, Raoult D. Stem-loop RNA hairpins in giant viruses: Invading rRNA-like repeats and a template free RNA. Front Microbiol. 2018; 10.3389/fmicb.2018.00101 29449833 PMC5799277

[2] Alexander RP, Fang G, Rozowsky J, Snyder M, Gerstein MB. Annotating non-coding regions of the genome. Nature Reviews Genetics. 2010 10.1038/nrg2814 20628352

[3] Ariel F, Romero-Barrios N, Jégu T, Benhamed M, Crespi M. Battles and hijacks: Noncoding transcription in plants. Trends in Plant Science. 2015 10.1016/j.tplants.2015.03.003 25850611

[4] Bernstein B, Birney E, Dunham I, Green E, Gunter C, Snyder M. An integrated encyclopedia of DNA elements in the human genome. Nature. 2012; citeulike-article-id:11191048\n 10.1038/nature11247 22955616 PMC3439153

[5] Preker P, Nielsen J, Kammler S, Lykke-Andersen S, Christensen MS, Mapendano CK, et al RNA exosome depletion reveals transcription upstream of active human promoters. Science (80-). 2008; 10.1126/science.1164096 19056938

[6] Seila AC, Calabrese JM, Levine SS, Yeo GW, Rahl PB, Flynn RA, et al Divergent Transcription from Active Promoters. Science (80-). 2008; 10.1126/science.1162253 19056940 PMC2692996

[7] Calin GA, Dumitru CD, Shimizu M, Bichi R, Zupo S, Noch E, et al Nonlinear partial differential equations and applications: Frequent deletions and down-regulation of micro- RNA genes miR15 and miR16 at 13q14 in chronic lymphocytic leukemia. Proc Natl Acad Sci. 2002; 10.1073/pnas.242606799 12434020 PMC137750

[8] Si ML, Zhu S, Wu H, Lu Z, Wu F, Mo YY. miR-21-mediated tumor growth. Oncogene. 2007; 10.1038/sj.onc.1210083 17072344

[9] Taft RJ, Glazov EA, Cloonan N, Simons C, Stephen S, Faulkner GJ, et al Tiny RNAs associated with transcription start sites in animals. Nat Genet. 2009; 10.1038/ng.312 19377478

[10] Watanabe T, Tomizawa SI, Mitsuya K, Totoki Y, Yamamoto Y, Kuramochi-Miyagawa S, et al Role for piRNAs and noncoding RNA in de novo DNA methylation of the imprinted mouse Rasgrf1 locus. Science (80-). 2011; 10.1126/science.1203919 21566194 PMC3368507

[11] DiGiacomo M, Comazzetto S, Saini H, DeFazio S, Carrieri C, Morgan M, et al Multiple Epigenetic Mechanisms and the piRNA Pathway Enforce LINE1 Silencing during Adult Spermatogenesis. Mol Cell. 2013; 10.1016/j.molcel.2013.04.026 23706823

[12] Hargreaves AD, Zhou L, Christensen J, Marlétaz F, Liu S, Li F, et al Genome sequence of a diabetes-prone rodent reveals a mutation hotspot around the ParaHox gene cluster. Proc Natl Acad Sci. 2017; 10.1073/pnas.1702930114 28674003 PMC5530673

[13] Lovell P V., Wirthlin M, Wilhelm L, Minx P, Lazar NH, Carbone L, et al Conserved syntenic clusters of protein coding genes are missing in birds. Genome Biol. 2014; 10.1186/s13059-014-0565-1 25518852 PMC4290089

[14] Blank A, Gallant JA, Burgess RR, Loeb LA. An RNA Polymerase Mutant with Reduced Accuracy of Chain Elongation. Biochemistry. 1986; 10.1021/bi00368a013 3098280

[15] Ninio J. Connections between translation, transcription and replication error-rates. Biochimie. 1991; 10.1016/0300-9084(91)90186-5 1805967

[16] Li M, Wang IX, Li Y, Bruzel A, Richards AL, Toung JM, et al Widespread RNA and DNA sequence differences in the human transcriptome. Science (80-). 2011; 10.1126/science.1207018 21596952 PMC3204392

[17] Bahn JH, Lee JH, Li G, Greer C, Peng G, Xiao X. Accurate identification of A-to-I RNA editing in human by transcriptome sequencing. Genome Res. 2012; 10.1101/gr.124107.111 21960545 PMC3246201

[18] Strathern JN, Jin DJ, Court DL, Kashlev M. Isolation and characterization of transcription fidelity mutants. Biochimica et Biophysica Acta—Gene Regulatory Mechanisms. 2012 10.1016/j.bbagrm.2012.02.005 22366339 PMC7511983

[19] Bar-Yaacov D, Avital G, Levin L, Richards AL, Hachen N, Rebolledo Jaramillo B, et al RNA-DNA differences in human mitochondria restore ancestral form of 16S ribosomal RNA. Genome Res. 2013; 10.1101/gr.161265.113 23913925 PMC3814879

[20] Knippa K, Peterson DO. Fidelity of RNA polymerase II transcription: Role of Rbp9 in error detection and proofreading. Biochemistry. 2013; 10.1021/bi4009566 24099331

[21] Zhou YN, Lubkowska L, Hui M, Court C, Chen S, Court DL, et al Isolation and characterization of RNA polymerase rpoB mutations that alter transcription slippage during elongation in Escherichia coli. J Biol Chem. 2013; 10.1074/jbc.M112.429464 23223236 PMC3554936

[22] Wang IX, Grunseich C, Chung YG, Kwak H, Ramrattan G, Zhu Z, et al RNA-DNA sequence differences in Saccharomyces cerevisiae. Genome Res. 2016; 10.1101/gr.207878.116 27638543 PMC5088596

[23] Bass BL. RNA Editing by Adenosine Deaminases That Act on RNA. Annu Rev Biochem. 2002; 10.1146/annurev.biochem.71.110601.135501 12045112 PMC1823043

[24] Schaub M, Keller W. RNA editing by adenosine deaminases generates RNA and protein diversity. Biochimie. 2002; 10.1016/S0300-9084(02)01446-3 12457566

[25] Chen C, Bundschuh R. Systematic investigation of insertional and deletional RNA-DNA differences in the human transcriptome. BMC Genomics. 2012; 10.1186/1471-2164-13-616 23148664 PMC3505181

[26] Park E, Williams B, Wold BJ, Mortazavi A. RNA editing in the human ENCODE RNA-seq data. Genome Res. 2012; 10.1101/gr.134957.111 22955975 PMC3431480

[27] Wang IX, Core LJ, Kwak H, Brady L, Bruzel A, McDaniel L, et al RNA-DNA differences are generated in human cells within seconds after RNA exits polymerase II. Cell Rep. 2014; 10.1016/j.celrep.2014.01.037 24561252 PMC4918108

[28] Lee SY, Joung JG, Park CH, Park JH, Kim JH. RCARE: RNA Sequence Comparison and Annotation for RNA Editing. BMC Med Genomics. 2015; 10.1186/1755-8794-8-S2-S8 26043858 PMC4460956

[29] Porath HT, Carmi S, Levanon EY. A genome-wide map of hyper-edited RNA reveals numerous new sites. Nat Commun. 2014; 10.1038/ncomms5726 25158696 PMC4365171

[30] Kumar S, Vo AD, Qin F, Li H. Comparative assessment of methods for the fusion transcripts detection from RNA-Seq data. Sci Rep. 2016; 10.1038/srep21597 26862001 PMC4748267

[31] Seligmann H. Overlapping genes coded in the 3’-to-5’-direction in mitochondrial genes and 3’-to-5’ polymerization of non-complementary RNA by an ‘invertase’. J Theor Biol. 2012; 10.1016/j.jtbi.2012.08.044 22995821

[32] Seligmann H. Polymerization of non-complementary RNA: Systematic symmetric nucleotide exchanges mainly involving uracil produce mitochondrial RNA transcripts coding for cryptic overlapping genes. BioSystems. 2013; 10.1016/j.biosystems.2013.01.011 23410796

[33] Seligmann H. Triplex DNA:RNA, 3′-to-5′ Inverted RNA and Protein Coding in Mitochondrial Genomes. J Comput Biol. 2013; 10.1089/cmb.2012.0134 23841652

[34] Warthi G, Seligmann H. Swinger RNAs in the Human Mitochondrial Transcriptome. In: Seligmann H, Warthi G, editors. Mitochondrial DNA-new insights. Chapter 4, 79–92. 10.5772/intechopen.80805

[35] Seligmann H. Systematic asymmetric nucleotide exchanges produce human mitochondrial RNAs cryptically encoding for overlapping protein coding genes. J Theor Biol. 2013; 10.1016/j.jtbi.2013.01.024 23416187

[36] Seligmann H. Translation of mitochondrial swinger RNAs according to tri-, tetra- and pentacodons. BioSystems. 2016; 10.1016/j.biosystems.2015.11.009 26723232

[37] Seligmann H. Swinger RNA self-hybridization and mitochondrial non-canonical swinger transcription, transcription systematically exchanging nucleotides. J Theor Biol. 2016; 10.1016/j.jtbi.2016.04.007 27079465

[38] Seligmann H. Mitochondrial swinger replication: DNA replication systematically exchanging nucleotides and short 16S ribosomal DNA swinger inserts. BioSystems. 2014; 10.1016/j.biosystems.2014.09.012 25283331

[39] Seligmann H. Sharp switches between regular and swinger mitochondrial replication: 16S rDNA systematically exchanging nucleotides A<->T+C<->G in the mitogenome of Kamimuria wangi. Mitochondrial DNA. 2016; 10.3109/19401736.2015.1033691 25865623

[40] Seligmann H. Species radiation by DNA replication that systematically exchanges nucleotides? J Theor Biol. 2014; 10.1016/j.jtbi.2014.08.036 25192628

[41] Seligmann H. Swinger RNAs with sharp switches between regular transcription and transcription systematically exchanging ribonucleotides: Case studies. BioSystems. 2015; 10.1016/j.biosystems.2015.07.003 26163926

[42] Seligmann H. Chimeric mitochondrial peptides from contiguous regular and swinger RNA. Comput Struct Biotechnol J. 2016; 10.1016/j.csbj.2016.06.005 27453772 PMC4942731

[43] Seligmann H. Codon expansion and systematic transcriptional deletions produce tetra-, pentacoded mitochondrial peptides. J Theor Biol. 2015; 10.1016/j.jtbi.2015.09.030 26456204

[44] Seligmann H. Natural mitochondrial proteolysis confirms transcription systematically exchanging/deleting nucleotides, peptides coded by expanded codons. J Theor Biol. 2017; 10.1016/j.jtbi.2016.11.021 27899286

[45] Seligmann H. Systematically frameshifting by deletion of every 4th or 4th and 5th nucleotides during mitochondrial transcription: RNA self-hybridization regulates delRNA expression. BioSystems. 2016; 10.1016/j.biosystems.2016.03.009 27018206

[46] Atkins JF, Loughran G, Bhatt PR, Firth AE, Baranov P V. Ribosomal frameshifting and transcriptional slippage: From genetic steganography and cryptography to adventitious use. Nucleic Acids Res. 2016; 10.1093/nar/gkw530 27436286 PMC5009743

[47] El Houmami N, Seligmann H. Evolution of nucleotide punctuation marks: From structural to linear signals. Front Genet. 2017; 10.3389/fgene.2017.00036 28396681 PMC5366352

[48] Arquès DG, Michel CJ. A complementary circular code in the protein coding genes. J Theor Biol. 1996; 10.1006/jtbi.1996.0142 8917736

[49] Michel CJ, Ngoune V, Poch O, Ripp R, Thompson JD. Enrichment of Circular Code Motifs in the Genes of the Yeast Saccharomyces cerevisiae. Life. 2017; 10.3390/life7040052 29207500 PMC5745565

[50] Michel CJ. The maximal C^3^ self-complementary trinucleotide circular code X in genes of bacteria, eukaryotes, plasmids and viruses. J Theor Biol. 2015; 10.1016/j.jtbi.2015.04.009 25934352

[51] Michel C. The Maximal C3 Self-Complementary Trinucleotide Circular Code X in Genes of Bacteria, Archaea, Eukaryotes, Plasmids and Viruses. Life. 2017; 10.3390/life7020020 28420220 PMC5492142

[52] Kung JTY, Colognori D, Lee JT. Long noncoding RNAs: past, present, and future. Genetics. 2013; 10.1534/genetics.112.146704 23463798 PMC3583990

[53] Dila G, Michel CJ, Poch O, Ripp R, Thompson JD. Evolutionary conservation and functional implications of circular code motifs in eukaryotic genomes. BioSystems. 2019; 10.1016/j.biosystems.2018.10.014 30367916

[54] Gonzalez DL, Giannerini S, Rosa R. Circular codes revisited: A statistical approach. J Theor Biol. 2011; 10.1016/j.jtbi.2011.01.028 21277862

[55] Fimmel E, Strüngmann L. Codon Distribution in Error-Detecting Circular Codes. Life. 2016; 10.3390/life6010014 26999215 PMC4810245

[56] Garzon R, Volinia S, Papaioannou D, Nicolet D, Kohlschmidt J, Yan PS, et al Expression and prognostic impact of lncRNAs in acute myeloid leukemia. Proc Natl Acad Sci. 2014; 10.1073/pnas.1422050112 25512507 PMC4284555

[57] Mercer TR, Neph S, Dinger ME, Crawford J, Smith MA, Shearwood AMJ, et al The human mitochondrial transcriptome. Cell. 2011; 10.1016/j.cell.2011.06.051 21854988 PMC3160626

[58] Arquès DG, Michel CJ. A circular code in the protein coding genes of mitochondria. J Theor Biol. 1997; 10.1006/jtbi.1997.0513 9441820

[59] Seligmann H. Natural chymotrypsin-like-cleaved human mitochondrial peptides confirm tetra-, pentacodon, non-canonical RNA translations. BioSystems. 2016; 10.1016/j.biosystems.2016.07.010 27477600

[60] Seligmann H. Unbiased Mitoproteome Analyses Confirm Non-canonical RNA, Expanded Codon Translations. Comput Struct Biotechnol J. 2016; 10.1016/j.csbj.2016.09.004 27830053 PMC5094600

[61] Seligmann H. Reviewing evidence for systematic transcriptional deletions, nucleotide exchanges, and expanded codons, and peptide clusters in human mitochondria. BioSystems. 2017 10.1016/j.biosystems.2017.08.002 28807694

[62] Jestin JL, Kempf A. Chain termination codons and polymerase-induced frameshift mutations. FEBS Letters. 1997 10.1016/S0014-5793(97)01422-1 9428624

[63] Crick FH, Griffith JS, Orgel LE. CODES WITHOUT COMMAS. PNAS. 1957 10.1073/pnas.43.5.416 16590032 PMC528468

[64] Klobutcher LA, Farabaugh PJ. Shifty ciliates: Frequent programmed translational frameshifting in euplotids. Cell. 2002 10.1016/S0092-8674(02)01138-8 12526802

[65] Ketteler R. On programmed ribosomal frameshifting: The alternative proteomes. Frontiers in Genetics. 2012 10.3389/fgene.2012.00242 23181069 PMC3500957

[66] Advani VM, Dinman JD. Reprogramming the genetic code: The emerging role of ribosomal frameshifting in regulating cellular gene expression. BioEssays. 2016 10.1002/bies.201500131 26661048 PMC4749135

[67] Lacan J. Michel CJ. Analysis of a Circular Code Model. J Theor Biol. 2001 10.1006/jtbi.2001.2416 11894988

[68] Ahmed A, Frey G, Michel CJ. Frameshift signals in genes associated with the circular code. In Silico Biol. 2007;7(2): 155–68. 17688441

[69] Michel CJ. Circular code motifs in transfer and 16S ribosomal RNAs: A possible translation code in genes. Comput Biol Chem. 2012; 10.1016/j.compbiolchem.2011.10.002 22129773

[70] El Soufi K, Michel CJ. Circular code motifs near the ribosome decoding center. Comput Biol Chem. 2015; 10.1016/j.compbiolchem.2015.07.015 26547036

[71] Michel CJ. Circular code motifs in transfer RNAs. Comput Biol Chem. 2013; 10.1016/j.compbiolchem.2013.02.004 23727957

[72] Fisher RA. Questions and answers #14. The American Statistician. 1948; 2 (5): 30–31.)

[73] Khanna M, Van Bakel H, Tang X, Calarco JA, Babak T, Guo G, et al A systematic characterization of Cwc21, the yeast ortholog of the human spliceosomal protein SRm300. RNA. 2009; 10.1261/rna.1790509 19789211 PMC2779666

[74] Harish A, Kurland CG. Mitochondria are not captive bacteria. J Theor Biol. 2017; 10.1016/j.jtbi.2017.07.011 28754286

[75] Seligmann H. Giant viruses as protein-coated amoeban mitochondria? Virus Res. 2018; 10.1016/j.virusres.2018.06.004 29913250

[76] Gao S, Tian X, Chang H, Sun Y, Wu Z, Cheng Z, et al Two novel lncRNAs discovered in human mitochondrial DNA using PacBio full-length transcriptome data. Mitochondrion. 2018; 10.1016/j.mito.2017.08.002 28802668

[77] Bazin J, Baerenfaller K, Gosai SJ, Gregory BD, Crespi M, Bailey-Serres J. Global analysis of ribosome-associated noncoding RNAs unveils new modes of translational regulation. Proc Natl Acad Sci. 2017; 10.1073/pnas.1708433114 29087317 PMC5699049

