# Supplementary figures and images for: Transcripts with systematic nucleotide deletion of 1-12 nucleotide in human mitochondrion suggest potential non-canonical transcription
Source: PLoS One. 2019 May 23;14(5):e0217356. doi: 10.1371/journal.pone.0217356 (PMC6532905; doi:10.1371/journal.pone.0217356)

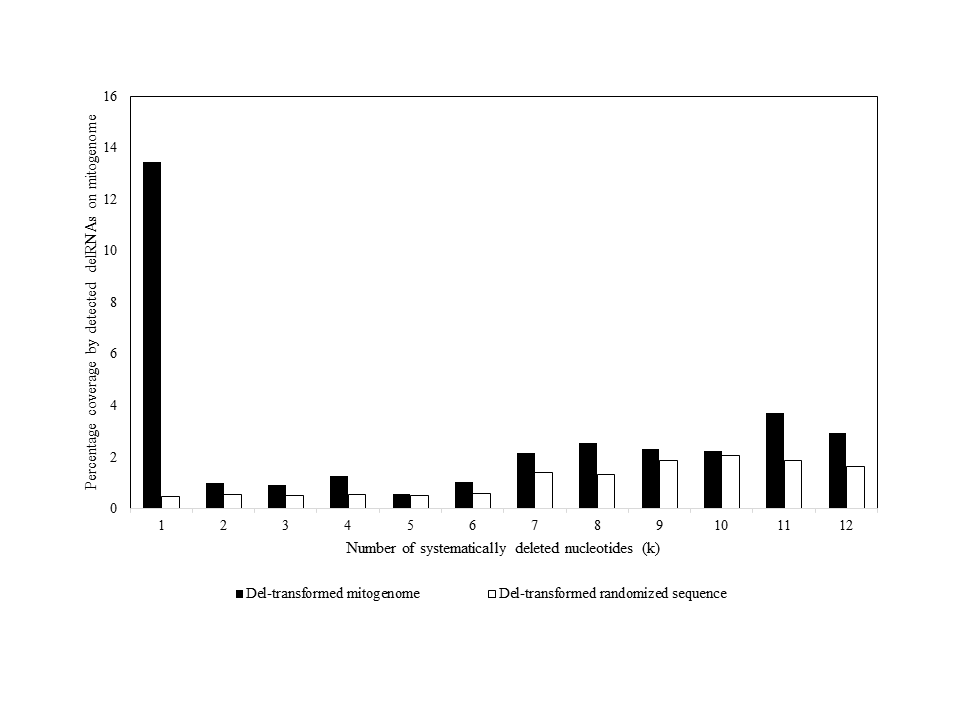

Supplement: S1 Fig — (TIF) [file pone.0217356.s001.tif]
